# Functional Real-Time Optoacoustic Imaging of Middle Cerebral Artery Occlusion in Mice

**DOI:** 10.1371/journal.pone.0096118

**Published:** 2014-04-28

**Authors:** Moritz Kneipp, Jake Turner, Sebastian Hambauer, Sandro M. Krieg, Jens Lehmberg, Ute Lindauer, Daniel Razansky

**Affiliations:** 1 Faculty of Medicine and Faculty of Electrical Engineering, Technische Universität München, Munich, Germany; 2 Institute for Biological and Medical Imaging, Helmholtz Zentrum München, Neuherberg, Germany; 3 Department of Neurosurgery, Klinikum rechts der Isar, Technische Universität München, Munich, Germany; 4 TUM-Neuroimaging Center, Technical University Munich, Munich, Germany; 5 Munich Cluster for Systems Neurology (SyNergy), Munich, Germany; National University of Singapore, Singapore

## Abstract

**Background and Purpose:**

Longitudinal functional imaging studies of stroke are key in identifying the disease progression and possible therapeutic interventions. Here we investigate the applicability of real-time functional optoacoustic imaging for monitoring of stroke progression in the whole brain of living animals.

**Materials and Methods:**

The middle cerebral artery occlusion (MCAO) was used to model stroke in mice, which were imaged preoperatively and the occlusion was kept in place for 60 minutes, after which optoacoustic scans were taken at several time points.

**Results:**

Post ischemia an asymmetry of deoxygenated hemoglobin in the brain was observed as a region of hypoxia in the hemisphere affected by the ischemic event. Furthermore, we were able to visualize the penumbra *in-vivo* as a localized hemodynamically-compromised area adjacent to the region of stroke-induced perfusion deficit.

**Conclusion:**

The intrinsic sensitivity of the new imaging approach to functional blood parameters, in combination with real time operation and high spatial resolution in deep living tissues, may see it become a valuable and unique tool in the development and monitoring of treatments aimed at suspending the spread of an infarct area.

## Introduction

Clinical and small animal imaging studies on stroke aim to further understanding into the development of the ischemic lesion [1], which in turn may lead to more effective treatments. Noninvasive functional imaging is central to these studies. However, imaging modalities commonly used for stroke visualization have generally high acquisition and running costs, with some also lacking the sufficient specificity, imaging speed, spatial resolution or penetration depth necessary for efficient investigations of disease progression in small animal models [1]–[3]. Multispectral optoacoustic tomography (MSOT) is a new imaging modality that utilizes generation of ultrasonic waves by wavelength-dependent absorption of ultrashort pulses of light in the imaged tissues [4]. Therefore, MSOT maintains the good functional contrast of the optical spectrum while also retaining significant advantages that are commonly associated with ultrasonic imaging, such as real-time operation and excellent spatial resolution unaffected by light scattering in deep tissues [5]. Being based on light absorption, optoacoustics can directly sense blood-related contrast, in particular concentration of deoxygenated hemoglobin (Hb) and oxygenated hemoglobin (HbO_2_), without introduction of contrast agents [6]. In addition, the ability to visualize real time kinetics of uptake and clearance of contrast agents, in particular in whole mouse brains, has been recently demonstrated with this method [7].

In this study, real-time MSOT imaging [8] was applied for longitudinal functional stroke imaging studies, aimed at identifying functional blood flow in the whole brain of a middle cerebral artery occlusion (MCAO) mouse model *in vivo.* Scans were made preoperatively for later comparison to post-operative scans and as a control. Infarct volume analysis was performed after the last *in-vivo* scan by means of histology after sacrificing the animal.

## Methods

### In-vivo MSOT Imaging

**Figure 1:**
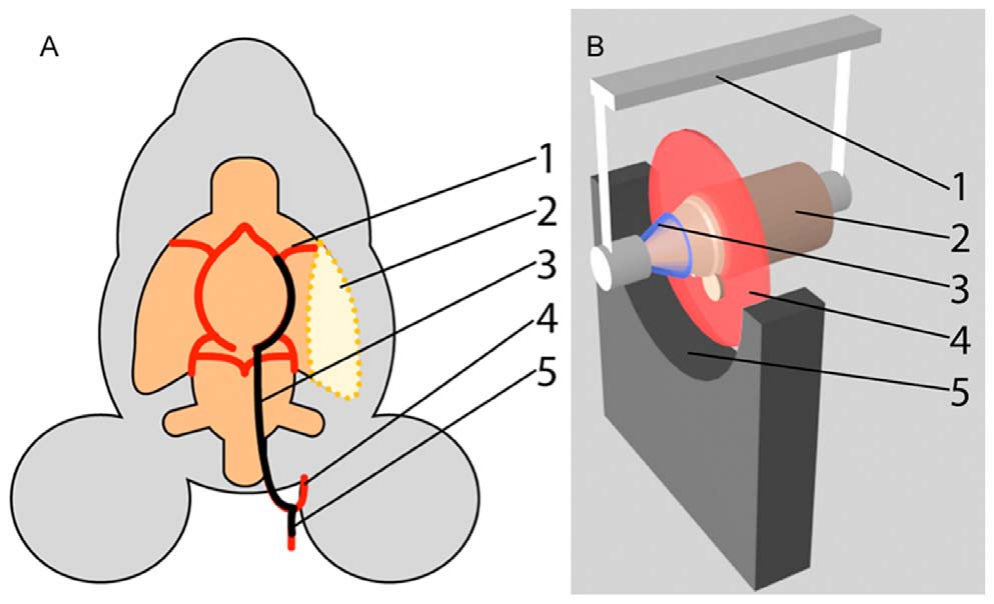
Illustrations of the procedure and experimental setup. A: Schematic representation of the MCAO procedure. The filament is forwarded into the MCA (1) through the ICA (3), starting at the bifurcation of the CCA (5) into the ECA (4) and ICA. This causes formation of the stroke volume (2). B: Schematic representation of the MSOT scanner. The mouse holder (1) secures the mouse (2) in the scanner and anesthesia is supplied through the gas mask (3). Optoacoustic signals are generated in the illumination plane (4) and detected by the transducer array (5), covering and angle of 172°.

The non-invasive optoacoustic scans were performed in vivo by acquiring whole-brain tomographical datasets formed from coronal slices imaged at 15 different excitation wavelengths between 710 and 850 nm. The system used was a cross-sectional small animal MSOT scanner, which uses a 64-element ultrasonic array for real-time 2D scanning [9]. MSOT offers high-resolution real-time imaging in deep tissues, combining advantages of high optical contrast and low scattering of ultrasound waves in biological tissues. The acquired broadband acoustic signals are generated through the optoacoustic phenomenon, whereby thermoelastic expansion in the target is created by irradiating it with nanosecond duration pulses of light from a tunable optical parametric oscillator laser [6]. In addition to the focused ultrasound transducer array, the MSOT scanner comprises an animal holder with a gas mask for anesthesia supply, and a motorized positioning stage to translate the animal relative to the illumination plane (Fig. 1B). Fiber bundles are distributed to form an illumination pattern that provides equal light fluence around the circumference of the imaged region. Ultrasonic coupling between the target and the transducer array is achieved by filling the acquisition chamber with water; the water is heated to 34°C to give a stable body temperature for the mice and a constant speed of sound in the coupling medium. The animal is further protected by a membrane, inhibiting direct contact between the coupling medium and the animal.

### Mouse Handling

All animals were treated in accordance with FELASA guidelines and the study was approved by local government (Regierung von Oberbayern, AZ 55.2-1-54-2531.2-6-10). This study saw seven mice (strain: Crl:SKH1-Hrhr, hairless, unpigmented, immunocompetent) operated upon and scanned; the mice were anesthetised with 1.5% isoflurane (Penlon vaporizer, UK) vaporized in pure oxygen; scans were made preoperatively for later comparison to post-operative scans and as a control.

Following the initial validation scan, the MCAO procedure [9] was performed outside the scanner. A small incision was made at the neck of the animal and the left external and internal carotid arteries (ECA, ICA) were freed from the surrounding tissue. A coated filament was introduced into the common carotid artery (CCA), advanced inside the ICA and secured to occlude the origin of the middle cerebral artery (MCA), as illustrated in Figure 1A.

During occlusion, a second *in-vivo* scan was performed. After 60 min of ischemia, the filament was retracted, the wound closed, and the animal allowed to survive for 24 hours. After 24 h, a final MSOT scan was performed under anesthesia.

### Data Analysis

The tomographic optoacoustic data recorded at each wavelength were reconstructed by interpolated model-matrix inversion [10]. The model-based framework allows for an accurate modeling of light and sound propagation in tissues as well as ultrasounic detector characteristics, thus most quantitative functional blood parameters can be extracted. Reconstruction artifacts due to the limited view of 172° of the transducer array are consequently minimized by including the array geometry in the reconstruction model. The optoacoustically-generated pressure distribution is given as.
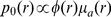
(1)where 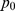 denotes the initially generated pressure at location 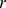, dependent upon the local light fluence 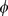 in J/m^2^ and the local absorption coefficient 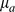 in cm^−1^. The reconstructed images are subsequently spectrally unmixed, using a linear regression method [4] in order to map distributions of Hb and HbO_2_ in the brain. For every data point in the reconstructed images, the algorithm uses least-squares to match the recorded optoacoustic spectrum to the known absorption spectra of Hb and HbO2. This results in distribution maps of the two states of hemoglobin, which are regarded as the dominant absorbers in biological tissue. Spectral unmixing is essentially performed on a per-pixel basis by solving the set of linear equations:
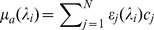
(2)where 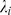 is the illumination wavelength 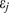 is the wavelength-dependent molar extinction coefficient of a particular chromophore 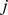 having a local concentration 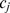 [11].

**Figure 2:**
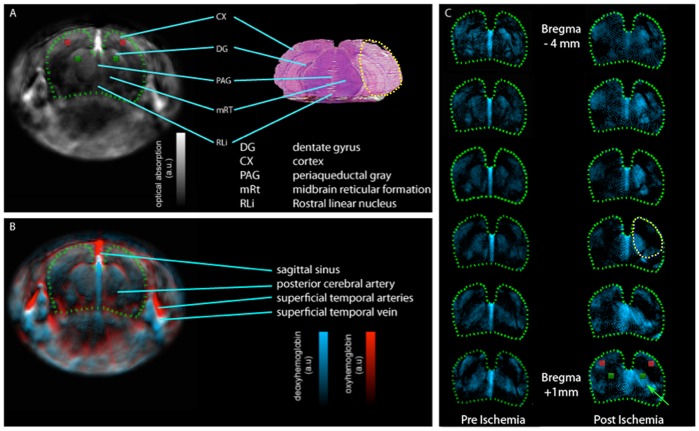
Whole-brain optoacoustic images of a mouse. A: Structural features visible in the cryosection (right) are matched with the corresponding optoacoustic image features (left). The representative stained cryosection shows the infarct area caused by 1 h MCAO followed by 24 h reperfusion. In the optoacoustic image, the area corresponding to the brain is enclosed by a green dotted line. The area affected by MCAO has been enclosed by a yellow dotted line. B: the multispectral data unmixed for the oxygenated and deoxygenated hemoglobin are overlaid on the single wavelength image from above (790 nm) in blue and red, respectively; the sagittal sinus is highly oxygenated due to the oxygen-isoflurane anesthetic. C: A coronal-slice set from a representative animal (distance between slices: 1 mm) with the multispectral unmixed data for deoxygenated hemoglobin in the brain presented in blue. The post ischemia set shows clear asymmetry (green arrow), while symmetry is intact in the pre ischemia set. Red and green squares in A and C indicate the ROIs for the ratio analysis.

Additional analysis using single wavelength images was performed. The isosbestic point of hemoglobin is close to 790 nm, thus signals recorded at this wavelength are closely related to the total cerebral blood volume (CBV). In order to analyze overall trends in the study, two areas were compared pre-, during, and post-ischemia at this wavelength. The comparison made was ratios of the mean value of a region of interest (ROI) in the ischemic cortex to the mean of its contralateral counterpart (marked by red squares in Figs. 2A and 2C); ratios were also taken for ROIs located in the striatum (marked by green squares in Figs. 2A and 2C). Percentage changes of the ratios relative to each pre-ischemia value were calculated. For statistical comparisons of absolute ratio data, the Friedman Repeated Measures Analysis of Variance on Ranks was performed. If a significant difference was detected, Tukey Test (all pairwise multiple comparison procedures) was used as post hoc analysis; p<0.05 was considered significant.

### Histological Validation

The animals were then sacrificed and their brains excised and frozen at −80°C. To validate the stroke area post mortem coronal brain slices were taken using a cryostat with two 10 µm sections being mounted on a glass slide every 500 µm. H&E staining was performed and the infarct volumes were analyzed.

## Results

A typical cross-sectional single wavelength optoacoustic image mainly reveals the major vessels and other anatomical features in the head. These correlate to some of the features that can be identified in the histological slices taken post mortem (Fig. 2A). However, the functional characteristics can be better visualized in the images spectrally unmixed for the presence of Hb and HbO_2_ (Fig. 2B). When comparing the hemoglobin Hb signal of the preoperative scans to that of the scans taken 24 hours later, a clear asymmetry throughout a large portion of the brain becomes visible, as can be seen in Figure 2C. The signal likely corresponds to part of the tissue still suffering from hypoperfusion after the stroke. The Hb signal did not correspond directly to the necrotic ischemic lesion found in the stained coronal slices, but rather to the immediate surroundings of the lesion. While the hemisphere is reperfused after the 60-minute occlusion time, the area adjacent to the ischemic lesion appears to experience further deficient perfusion [12], resulting in the Hb-signal asymmetry after 24 hours.

**Figure 3:**
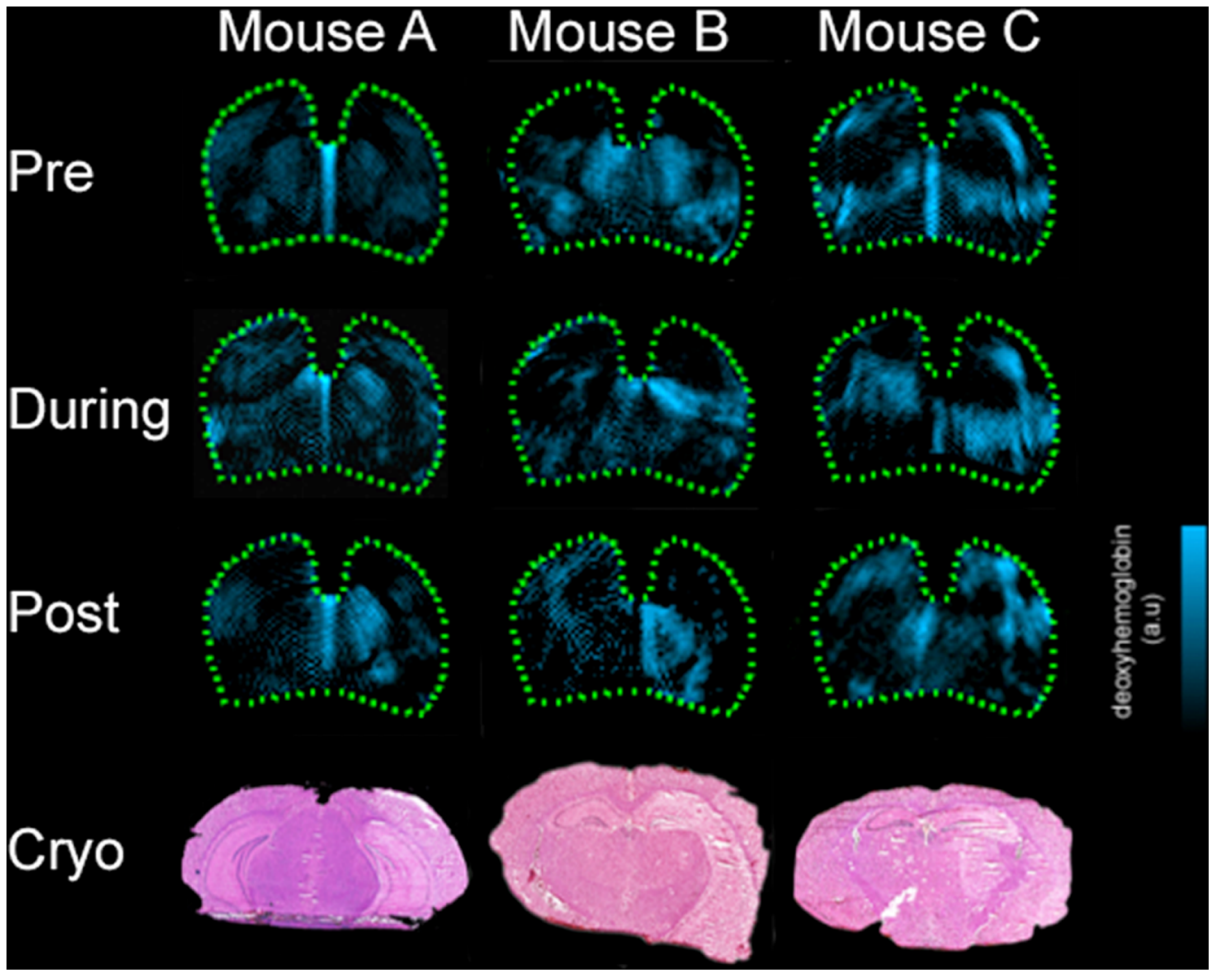
Deoxygenated hemoglobin distribution in several mice. From top down: Pre-, during, and 24 hours post-ischemia MSOT images for three MCAO mice (left to right) with corresponding H&E stained cryosections for each mouse (bottom; per mouse). MSOT: multispectral data for deoxygenated hemoglobin are presented in blue. Compared with the pre-ischemia situation, images taken during and 24 h post ischemia show clear asymmetry in the vicinity of the infarcted regions, as also apparent in the corresponding histological sections.

Similar asymmetry is visible during the ischemia, as can be seen in Fig. 3. It is hypothesized that this hemodynamically compromised area represents part of the ischemic penumbra, which consists of hypoperfused but still viable tissue [13]. Even 24 h after the onset of stroke this region is not completely destroyed but may, depending on the course of the stroke, recover or become necrotic [14]. As can be further observed in Fig. 3, the Hb signal varies in strength and spatial distribution from mouse to mouse. While Mouse A exhibited the largest volume of increased Hb, mice B and C showed smaller areas, as can also be verified via the corresponding histological slices (infarct volumes at 24 h as analyzed from the histological sections: mouse A: 58.5 mm^3^, mouse B: 49 mm^3^, mouse C: 48.5 mm^3^). The size of the area of Hb-asymmetry and the associated signal strength therefore appear to be closely related to the histological infarct volume. Some mice appear to show little or no Hb signal in the cortex even after reperfusion (mouse A and B in Fig. 3). The infarct core has no (or dramatically reduced) cellular activity and thus limited oxygen consumption without the need to deoxygenate the present hemoglobin, resulting in pseudo-normal or reduced Hb signal [14].

**Figure 4:**
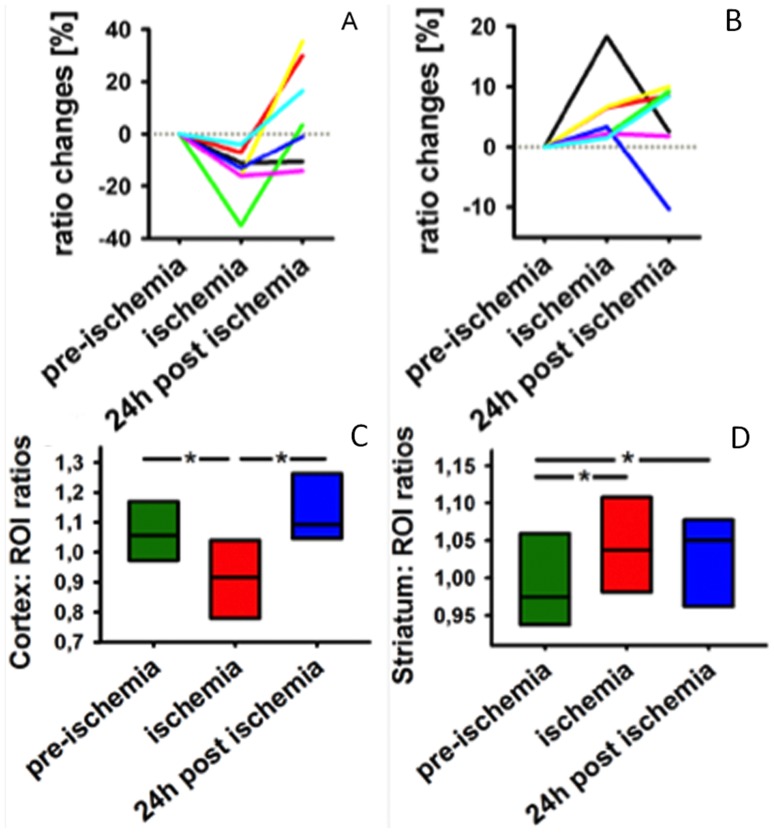
CBV ratio analysis for all the 7 imaged animals based on single wavelength optoacoustic images acquired at isosbestic wavelength of blood at 790 nm. Ratios of the mean in the ischemic cortex to the mean of its contralateral counterpart were calculated for the cortical areas (marked by red squares in Figs. 2A and 2C) and striatum areas (marked by green squares in Figs. 2A and 2C) and % changes relative to pre-ischemia values are presented. A: All mice show reduction in CBV in the cortex, followed by return to normal values after reperfusion at 24 h (middle). B: For the striatum areas, the mice show an increase of CBV during ischemia, which remains elevated in most of the animals after reperfusion. Averaged data of absolute ratio values from all the mice (box plots with median and 25^th^ and 75^th^ percentile) are shown in panels C and D for the cortical and striatum areas, respectively.

During artery occlusion, the total CBV in the ischemic core located in the cortex is reduced (Fig. 4A). This reduction in CBV results in a reduced ROI-ratio in all mice. At 24 h following reperfusion, the ratios have returned to nearly normal values. The averaged data of all 7 mice show that a significant difference exists between ratios during and 24 h post- ischemia (illustrated by the asterisks in Fig. 4C). No difference exists between pre- and 24 h post-ischemia. For the ROIs located in the striatum, a different trend is apparent (Figure 4B). While the ratios increase during artery occlusion for all mice, they further remain elevated also at 24 h following reperfusion. Overall, the averaged data for all 7 mice show a significant difference between the ischemic and post-ischemic periods as compared to pre-ischemia conditions (Fig. 4D).

## Discussion

The current study investigated applicability of real-time multispectral optoacoustic tomography (MSOT) for the monitoring of stroke progression following MCAO in the whole brain of living mice. The *in vivo* optoacoustic scans of the whole animal head revealed the major vessels and their functional oxygenation parameters. Post ischemia an asymmetry of deoxygenated hemoglobin in the brain was observed as a region of hypoxia in the hemisphere affected by the ischemic event. This region was mainly located in the border zone adjacent to necrotic tissue and was present at 24 h post occlusion. Since similar asymmetry is found during the ischemia this hemodynamically compromised area is hypothesized to be part of the hypoperfused, but still viable, ischemic penumbra. Histological findings further confirmed that the size of the area of Hb-asymmetry and the associated signal strength, as revealed in the *in-vivo* optoacsoustic scans, are closely related to the infarct volume.

The ratios between hemispheres demonstrate different longitudinal behavior in different areas of the brain. While cortex ratios dropped during ischemia for all mice, returning to nearly normal values after reperfusion, similar ratios in the striatum areas increased for all mice during artery occlusion and remained elevated at 24 h following reperfusion. It was previously confirmed that, due to the less pronounced reduction of blood flow in the penumbral region, autoregulatory dilation of the local cerebral circulation leads to an increase of CBV [15]. In the case that blood flow is sufficient, the cells located in the hypoxic penumbra will maintain metabolism and survive the occlusion; where blood flow is insufficient to support cell activity over time, the area will become part of the infarct.

Indeed, the investigation into potential treatments for stroke necessitates non-invasive longitudinal studies. Here, through non-invasive functional MSOT imaging, it was possible to visualize the penumbra as a localized hypoxic area in the brain. Potential future studies could investigate the how different treatments affect the development of this hypoxic area, and thus limit the spread of the necrotic core. From a technical perspective, it would be ideal to simultaneously capture true three-dimensional MSOT data from the entire brain, which is a subject of our ongoing investigations. This would further allow achieving isotropic resolution in the imaged region, reduce out-of-plane image artifacts and lead to overall improvement of the data quantification abilities.

## Conclusions

In summary it has been shown that, although the ischemic lesion was not directly detectable using MSOT, an adjacent localized region with characteristic hemodynamic disturbance was detected throughout the brain that most certainly reflects the ischemic penumbra. The unique capacity to monitor the penumbra development non-invasively and longitudinally without the introduction of contrast agents demonstrates further potential for investigating the ability of acute stroke therapies aimed at limiting the lesion size. Finally, the intrinsic sensitivity of MSOT to functional blood parameters, in combination with real time operation and high spatial resolution in deep tissues, may see it become a valuable and unique tool in functional stroke studies.
